# Are traumatic bilateral adrenal injuries associated with higher morbidity and mortality?-A prospective observational study

**DOI:** 10.1186/s13032-015-0026-1

**Published:** 2015-08-06

**Authors:** Ananya Panda, Atin Kumar, Shivanand Gamanagatti, Ashu Seith Bhalla, Raju Sharma, Subodh Kumar, Biplab Mishra

**Affiliations:** Departments of Radiology, Jai Prakash Narayana Apex Trauma Centre, All India Institute of Medical Sciences, Ansari Nagar, New Delhi, 110029 India; Departments of Surgery, Jai Prakash Narayana Apex Trauma Centre, All India Institute of Medical Sciences, Ansari Nagar, New Delhi, 110029 India

## Abstract

**Background:**

Traumatic bilateral adrenal injuries are uncommon. Adrenal injuries are overall associated with worse outcome than non-adrenal injuries. However, direct comparative evidence between unilateral and bilateral adrenal injuries is unavailable in literature. This study aims to investigate clinical significance of bilateral adrenal hematomas in terms of injury severity, morbidity and mortality.

**Methods:**

All blunt trauma abdomen patients with adrenal gland involvement on initial CECT scans of abdomen presenting to our Level 1 trauma centre over 21 months were prospectively included and followed-up. Patients were divided into unilateral and bilateral adrenal hematoma groups. For all patients, mechanism of injury, initial pulse, blood pressure, respiratory rate, Glasgow Coma Scale (GCS) scores, Injury Severity Score (ISS), New Injury Severity Score (NISS), length of ICU stay (LOI), length of hospital stay (LOS), total blood products (TBP) received were recorded. Final outcome was noted as complete recovery; discharge with poor prognosis and death. Quantitative parameters between both groups were compared using appropriate statistical tests and P < 0.05 was considered significant.

**Results:**

Forty seven patients were detected to have adrenal hematomas, 34 with unilateral (30 right and 4 left) and 13 with bilateral involvement. An oval mass replacing the gland was the most common appearance of injury (35/60) and periadrenal fat stranding was most common associated finding (47/60). Patients with bilateral adrenal hematoma had significantly lower GCS (13 vs 15, P < 0.01), ISS (38 vs 22, P < 0.01), NISS (47 vs 21, P < 0.01), LOI as proportion of LOS (91.7 % vs 10.5 %, *P* = 0.01) and TBP received (10 vs 4 units, P < 0.05). Outcome in bilateral group was comparatively worse with higher proportion of deaths or discharge with poor prognosis (*P* = 0.000).

**Conclusions:**

Patients with bilateral adrenal injury are associated with higher injury severity, morbidity and mortality compared to unilateral adrenal injury.

## Background

The adrenals are bilateral small inverted V or Y shaped glands situated in the retroperitoneum. Since the adrenal glands are small and well protected by surrounding organs like kidneys, liver, spleen, stomach and spine, traumatic adrenal hemorrhage is relatively uncommon. But simultaneously, due to their sheltered location, adrenal hemorrhage has been noted in more severe trauma [1, 2]. The reported incidence of adrenal trauma is variable, ranging from 0.15 to 4.95 % with a similar incidence in both adults and children [1–7]. Traumatic bilateral adrenal hemorrhage is still rarer with literature being confined to few case reports and limited case series [8–11].

Computed Tomography (CT) is the modality of choice for evaluation of adrenal trauma and CT appearances of adrenal injuries have been previously enumerated in literature [3, 4, 7, 12–16]. Adrenal hematomas per se have also been reported to be associated with higher injury severity and mortality compared to non-adrenal gland trauma. The exact cause of the higher mortality and morbidity associated with adrenal gland trauma is unknown but adrenal insufficiency has been hypothesized to play some role [9, 11, 17–19]. The clinical significance of bilateral adrenal hematoma remains even more unknown due to the relative rarity of bilateral adrenal hematomas and the insufficient number of cases with bilateral adrenal hematomas among patients with adrenal injuries.

Thus the purposes of the study were to evaluate adrenal hematomas with Multidetector CT (MDCT) technology and investigate the clinical significance of bilateral traumatic adrenal hematomas as compared to unilateral adrenal hematomas in terms of injury severity, morbidity and mortality.

## Methods

This was a prospective observational study conducted in our Level 1 apex Trauma Centre over a duration of 21 months from January 2011 to September 2012 after obtaining due approval from Institutional Ethics Committee. During this period, the contrast enhanced CT (CECT) scans of 1238 patients with blunt trauma abdomen obtained at the time of presentation to the hospital were evaluated for presence of adrenal injury. The patients were recruited into either unilateral or bilateral groups on basis of CT evidence of adrenal injury. Thereafter these patients were prospectively followed up clinically during their intra-hospital stay till final outcome as either death or discharge.

### CECT protocol and analysis of adrenal injury

Thoracoabdominal CECT scans were performed either on 40-detector (Somatom Sensation, Siemens, Erlangen, Germany) or 64-detector MDCT scanner (Definition AS, Siemens, Erlangen, Germany). The scans were acquired in portal venous phase, 60—70 s after hand-injection of 100 ml of non-ionic low-osmolar iodinated contrast (Iomeprol, Iomeron®400, Bracco, s.p.a, Milan, Italy), injected approximately at rate of 3–4 ml/s. The scanning parameters were collimation and pitch of 24 x1.2 and 1.4 on the 40-detector scanner and 16 x 1.2 and 0.8 on the 64-detector scanner respectively. The CT scans were analysed on the 3D workstation in axial, coronal and sagittal planes to evaluate adrenal injury.

**Fig. 1:**
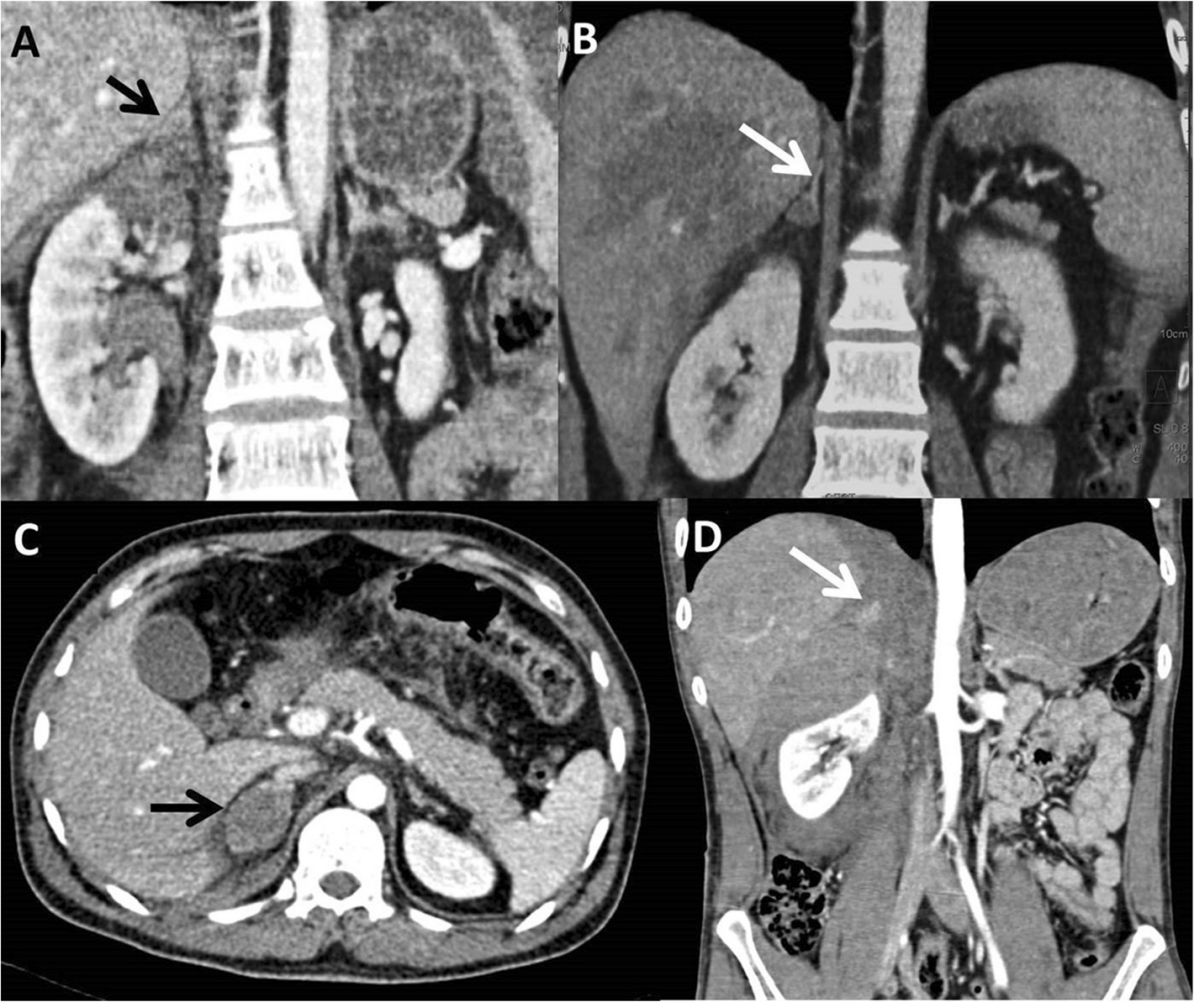
**a**-**d**: Various CT appearances of adrenal injuries. CT images show bulky right adrenal gland (**a**), hematoma splaying limbs (**b**), oval mass replacing gland (**c**) and active contrast extravasation in right adrenal gland hematoma (**d**)

Adrenal injury was said to be present if any of the following previously described appearances [4, 12, 14, 15] were noted on CT. These included a) bulky gland with ill-defined limbs; with or without adjacent fat stranding, b) triangular or oval hematoma with splaying of limbs. In this type, at least one or both limbs were visualised and referred to a focal intraparenchymal or intramedullary haemorrhage c) well-defined oval or round hematoma replacing the whole gland with non-visualisation of both limbs. This represented a diffuse hematoma involving the whole gland and d) hematoma with active extravasation (Fig. 1). For hematomas, maximum size and attenuation value was calculated as a mean of three attenuation values obtained by placing similar Regions of Interests (ROI) on largest portion of the hematoma. For all adrenal injuries, periadrenal stranding was noted as either present or absent. Also diaphragmatic crus thickening was subjectively noted as present or absent.

In those study patients requiring repeat CT scans as a part of their in-hospital treatment, the repeat scans were also evaluated for appearances of adrenal injuries.

### Clinical and Laboratory data analysis

For all patients detected to have adrenal injury, mechanism of injury, pulse, blood pressure, respiratory rate, Glasgow Coma Scale (GCS) scores and laboratory parameters such as haemoglobin, haematocrit, blood urea, creatinine were noted at the time of presentation. The total extent and severity of injuries were also noted based on the combination of clinical examination and radiological examinations. These injury descriptions were used to calculate Injury Severity Score (ISS) and New Injury Severity Score (NISS). These scores are internationally accepted and validated scores for objective assessment of trauma patients among different populations and studies.

During admission, morbidity parameters such as the length of ICU stay (LOI), total length of hospital stay (LOS), LOI as a proportion of LOS (LOI/LOS) and total blood products (TBP) [sum of units of red blood cells, platelets, fresh frozen plasma (FFPs)] transfused were recorded.

The final outcome was noted as complete recovery and discharge; discharge with poor prognosis/near-vegetative state and death. For patients who died, the cause of death was determined from hospital records. The term “poor prognosis/near-vegetative state” was used for patients who were completely bed-ridden and unable to independently perform activities of daily living.

### Statistical analysis

Statistical analyses were done retrospectively at the end of the study period. The radiological descriptions of injury were tabulated. The clinical, laboratory and outcome parameters were grouped into unilateral or bilateral involvement and compared using Mann-Whitney’s test and Fischer’s exact test and P-value < 0.05 was considered significant.

## Results

Forty-seven patients were detected to have adrenal injuries on CT, of which 34 were unilateral (30 right sided and 4 left sided) and 13 were bilateral. A total of 60 adrenal injuries were radiologically analysed, 43 on the right and 17 on left side. The mean age of overall adrenal trauma population was 32 years (5–52 years). The most common mechanism of injury was road traffic accident (RTA) seen in 66 % (31/47) while fall from height was seen in 23.4 % of patients (11/47). Fall of heavy object on abdomen was noted in 4/47 while 1 patient was a case of assault.

The most common associated abdominal injury in overall population was liver injury seen in 46.8 % (22/47) followed by splenic injury seen in 10/47 patients (21.3 %). The associated abdominal and extra-abdominal injuries in the study population have been tabulated in Table 1. Only two patients (both with right adrenal hematomas) had isolated adrenal injuries without any other associated intra or extra-abdominal injuries. One patient was a 18-year old man with direct assault to the abdomen by sticks, while the other patient was a 23 year old cyclist hit by a four-wheeler car (road traffic accident).

**Table 1:** Distribution of abdominal and extrabdominal injuries in overall adrenal trauma population (*n* = 47)

Abdominal Injuries	No of patients (%)	Extra-abdominal Injuries	No of patients (%)
Liver	22 (46.8)	Chest wall/ lungs	29 (61.7)
Spleen	10 (21.3)	Pelvis	16 (34.04)
Kidneys	5 (10.6)	Spine	16 (34.04)
Pancreas	3 (6.4)	Head	14 (29.8)
Bowel/ mesentery	4 (8.5)	Extremities	13 (27.7.)
		Face	7 (14.9)

Numbers in parenthesis indicate percentages

### Radiological evaluation

**Fig. 2:**
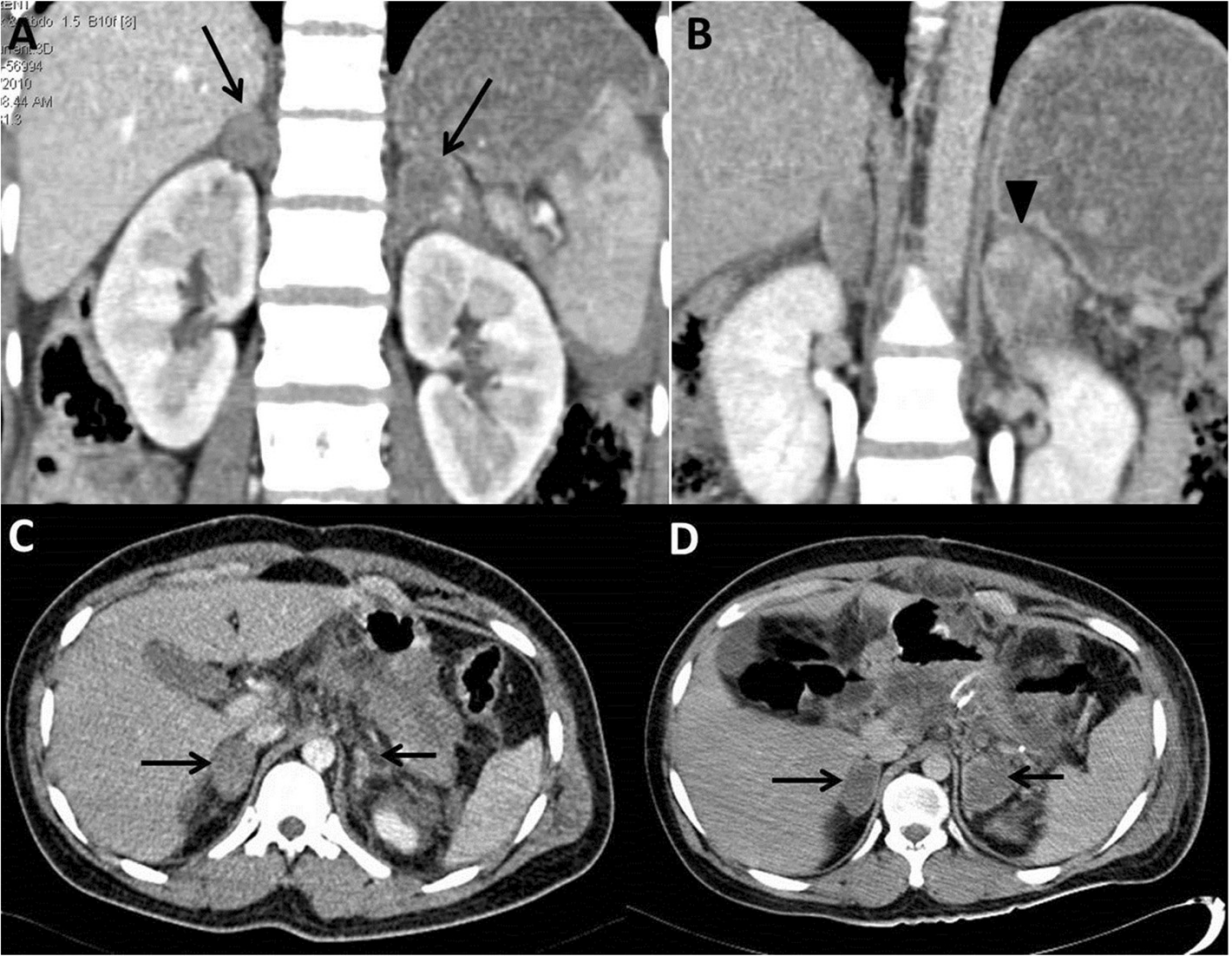
Bilateral adrenal injuries. CT coronal sections (**a**,**b**) show bilateral adrenal hematomas (arrows, **a**) with active extravasation of contrast in the left adrenal gland on delayed scan (arrowhead, **b**). Due to persistent hypotension, intraoperative packing of left adrenal gland with surgigel was done but patient died within 48 h due to intractable hypotension. CT axial sections (**c**,**d**) of another patient show right oval hematoma and left bulky gland on day 1 CT (arrows, **c**). On follow-up day 10 CT, the left adrenal injury evolved into an oval hematoma (arrows, **d**)

Out of 47 patients with CT evidence of adrenal injury in 60 adrenal glands, the most common CT appearance of adrenal injury was an oval hematoma replacing the whole adrenal gland seen in 35/60 hematomas followed by intraparenchymal hematomas seen in 15/60 hematomas. Bulky adrenal glands were seen in 8/60 hematomas while active extravasation was seen in 2/60 hematomas. One patient with bilateral adrenal hematoma had active extravasation from left adrenal gland in whom surgery was done and packing of the bleeding site with surgigel (sponge) was attempted (Fig. 2a,b).

The mean size of adrenal hematomas were 3.29 cms (1.0- 10.6, SD = 1.42 cms) and the mean attenuation value of adrenal hematomas was 42.69 HU (14–72, SD = ± 13.16 HU). Among the associated CT findings, periadrenal stranding was seen in 78.3 % (47 out of 60 hematomas) and crus thickening was seen in 48.3 %% (29 out of 60 hematomas).

Five patients in unilateral group and two patients in bilateral group underwent repeat CT scans, performed variably from days 5 to 11 of admission. In one patient from bilateral group, evolution of injury was seen as left bulky, irregularly attenuating adrenal on day 1 CT evolved into an oval mass replacing the left gland on day 10 CT (Fig. 2c,d). In the other 6 patients, the morphological appearances of injury were similar but were more well-defined and hypoattenuating compared to earlier scans.

### Comparison between unilateral and bilateral groups

The median clinical parameters at presentation of both unilateral and bilateral adrenal hematomas groups have been tabulated in Table 2, There was a significant difference in GCS score between two groups (13 vs 15, *P* = 0.003).

**Table 2:** Comparison of clinical parameters at presentation between unilateral and bilateral groups

Parameters	Unilateral adrenal injury (*n* = 34)	Bilateral adrenal injury (*n* = 13)
	Median (Min-Max)	Median (Min-Max)
RR (cycle/min)	18 (6–34)	20 (16–24)
Pulse (beats/min)	78 (63–199)	100(78–164)
SBP (mm of Hg)	110 (80–160)	118 (90–178)
DBP (mm of Hg)	73 (58–110)	72(60–108)
GCS^a^	15 (3–15)^a^	13 (3–15)^a^
Hb (g/dl)	9.9 (3.4-15.5)	10.3 (6–14.5)
Hct	31.4 (10–48)	31.4 (17–41.8)
B.Urea (mg/dl)	29 (15–145)	30.5 (21–64)
Creat (mg/dl)	0.8 (0.3-10.6)	0.9 (0.6-1.2)

RR: Respiratory rate, SBP: Systolic BP, DBP: Diastolic BP, GCS: Glasgow Coma Scale, Hb: Hemoglobin. Hct: Hematocrit, B.urea: Blood Urea, Creat; Serum Creatinine

^a^Significant, P-value = 0.003 by Mann–Whitney test

In unilateral adrenal injury group, the median ISS and NISS were 22 (5–75) and 27 (5–61) respectively while in bilateral adrenal injury group, the median ISS and NISS were 38 (26–57) and NISS was 41 (34–57) respectively and the difference was significant (P-value = 0.003 by Mann–Whitney test). Thus bilateral adrenal hematomas were associated with significantly higher overall injury severity scores.

The median number of associated intra-abdominal organ injuries in bilateral adrenal injury, i.e. 4 organs was significantly higher than unilateral injuries (2 organs) (P-value = 0.003). Among extra-abdominal injuries, patients in bilateral group had significantly higher head and chest (lung/chest wall) injuries while there was no significant difference in incidence of facial, extremity and pelvic fractures and spine injuries in both groups. Also 6 patients in unilateral group had no extra-abdominal injuries in contrast to bilateral group wherein all patients had at least one extra-abdominal injury (P < 0.0001) (Table 3).

**Table 3:** Distribution of extra-abdominal injuries in unilateral and bilateral groups

Injury site	Unilateral adrenal injury (*n* = 34)	Bilateral adrenal injury (*n* = 13)	P-value (2 sample z-test)
Head	17.7 % (6/34)	61.5 % (8/13)	0.003
Facial fractures	11.8 % (4/34)	23.1 % (3/13)	0.331
Chest (Lung/ chest wall)	50 % (17/34)	92.3 % (12/13)	0.007
Extremity fractures	20.6 % (7/34)	46.2 % (6/13)	0.079
Pelvis	32.4 % (11/34)	38.5 % (5/13)	0.693
Spine	32.4 % (11/34)	38.5 % (5/13)	0.693
No extra-abdominal injury	17.5 % (6/34)	0.00 % (0/13)	<0.0001

Figures in parenthesis indicate numbers of patients

During hospital stay, while there was no significant difference in total length of hospital stay (LOS) and ICU stay (LOI) in both groups, LOI as a proportion of LOS in bilateral adrenal hematomas was significantly higher, indicating that patients with bilateral adrenal hematomas spent a significantly more time in critical care during their course of hospital admission (Table 4). Also patients with bilateral adrenal hematomas had received higher blood products secondary to underlying hemodynamic instability and coagulopathy.

**Table 4:** Comparison of morbidity parameters during course of hospital stay between unilateral and bilateral hematomas

Parameters	Unilateral adrenal hematoma (*n* = 34)	Bilateral adrenal hematoma (*n* = 13)
	Median (Min-Max)	Median (Min-Max)
Length of ICU stay (LOI)	2 (0–42)	8 (0–73)
Length of hospital stay (LOS)	10 (1–80)	9(0–75)
LOI/LOS (%)^a^	10.53 (0–100)	91.67 (0–100)
Total blood products (RBCs + Platelets + FFPs)^b^	4 (0–69)	10(1–45)

RBC: Red blood cells, FFP: Fresh Frozen Plasma, ^a^Significant, P-value = 0.01, Mann–Whitney test

^b^Significant, P-value = 0.04, Mann–Whitney test

Clinically, ten patients in bilateral adrenal group had refractory or repeated episodes of hypotension during their hospital stay. Among these, five patients had persistent hypotension and died within 24–72 h of admission due to cardiopulmonary arrest despite steroids and ionotropes admission. The other five patients had repeated episodes of hypotension and were also kept on intermittent steroids and ionotropic support and electrolyte supplementation. Among these five, one patient had one episode of cardiac arrest on day 5 and died on day 13. The remaining four were sent home after prolonged hospital stay with poor prognosis.

Among patients in unilateral group, repeated episodes of hypotension were clinically documented in two patients, both with severe spine and head injuries; one among these two died while the other patient was sent home with poor neurological prognosis. There were no documented episodes of hypotension in remaining patients including the four with multiple extra-adrenal injuries who died in unilateral group.

Thus the overall outcome in bilateral adrenal hematomas was significantly worse as only 3 out of 13 patients recovered and were discharged home in a stable state as compared to 28 out of 34 patients in unilateral group. There was a higher proportion of deaths or discharge with poor prognosis in patients with bilateral hematoma than unilateral (P-value = 0.000) (Table 5).

**Table 5:** Comparison of outcome between unilateral and bilateral hematomas

Outcome	Unilateral adrenal hematoma (*n* = 34)	Bilateral adrenal hematoma (*n* = 13)
	No of patients	No of patients
Stable at discharge	28	3
Death	5	6
Discharge with poor prognosis	1	4
Total	34	13

Significant, P-value = 0.000 by Fischer’s exact test

## Discussion

In our study, blunt adrenal trauma was noted in 47 out of 1238 patients over a 21 months period with an incidence of 3.8 % in blunt abdominal trauma which is consistent with the overall reported incidence of 0.15 % -4.95 % in literature [1–7]. However we observed a relatively high incidence of bilateral adrenal hematoma in our study population, as it was seen in 13 out of 47 patients with adrenal hematomas. Such a large representation of bilateral adrenal hematoma patients has not been reported in literature so far. In the published series till date, bilateral adrenal injuries were seen in 1 of 82 patients by Schwartz et al. [7] and 3 of 51 patients by Rana et al. [4]. In the series by Mehrazin et al. [2], 130 patients over a period of 15 years were identified to have adrenal hematoma, of which only 6 had bilateral involvement. The highest incidence was reported by Burks et al. [3] who found 3 bilateral hematomas in 20 patients studied over a 32-month period. The high incidence of bilateral adrenal hematoma in our study can possibly be attributed to high volume of cases referred to our trauma centre and also because we routinely perform CT scans in all patients admitted to our trauma centre with either Focussed Assessment with Sonography in Trauma (FAST) positive status or having a severe mechanism of injury (i.e. road traffic accident, fall from height, blunt trauma to chest or abdomen) irrespective of their FAST positive or negative status.

Among the unilateral adrenal hematoma patients, right sided adrenal hematomas were more common than on left side, similar to previously reported series [2–7]. The right sided predominance has been attributed to the direct transmission of increased venous pressure of inferior vena cava into right adrenal vein leading to congestion and rupture and also due to the greater compression of the right adrenal gland between the liver and the spine [20].

Nearly all patients in our study had associated intra-abdominal, thoracic, chest wall or orthopaedic injuries. Only two patients had isolated right adrenal injury without any other intra or extra-abdominal injury. The most common associated injuries were lung, liver and chest wall injuries which are similar to those reported by other authors [2–5, 12, 14, 15].

Radiologically, an oval mass replacing the entire adrenal gland was the most common appearance in our study, similar to previous reports [4, 12, 15, 16]. Periadrenal fat stranding was seen in 47 out of 60 hematomas (78.3 %) which is also similar to 61 %-93 % incidence reported previously by various authors [3, 4 13]. Similarly ipsilateral crus thickening was overall noted in 48.3 % (29/60 hematomas), comparable to incidence of 39- 61 % in previous studies [3, 14].

However, there were significant differences in the clinical, laboratory, morbidity and outcome data with respect to laterality. As described in Tables 1, 2, 3 and 4, patients with bilateral adrenal hematomas had significantly lower GCS, higher ISS and NISS, spent a larger proportion of their hospital stay in ICU requiring round-the clock critical monitoring and greater amount of intravenous fluids and blood products. ICU stays in these patients with bilateral hematomas were also marked by multiple episodes of hypotension, requiring inotropic support, steroid administration and coagulopathy requiring FFPs and platelets infusions. The overall outcome was also worse in bilateral adrenal hematomas as only 3 out 13 patients fully recovered. While various authors have described that adrenal injury is per se associated with higher mean ISS scores, lower GCS scores, higher morbidity and mortality as compared to non-adrenal trauma population [1, 4], such a head-to-head comparison between unilateral and bilateral groups is not available in literature for comparison.

However, whether this difference in outcome in our study can be directly attributed to adrenal insufficiency as a consequence of bilateral hematomas remains debatable. Previous anecdotal case reports have documented adrenal insufficiency in the form of hyperkalemia and hypotension in patients with traumatic bilateral adrenal haemorrhage that responded well to glucocorticoids [9, 11, 18, 19]. While adrenal insufficiency could have played a role as more patients in bilateral adrenal injuries group had refractory or repeated episodes of hypotension, these patients also had more severe injuries which could have also contributed to the worse outcome. Adrenal glands are usually well-cushioned from impact and severe force of injury is required to induce adrenal injury. To produce bilateral adrenal injuries, the force presumably needs to be even greater which would be associated with greater extent and severity of injuries. This is reflected by the higher ISS and NISS scores and lower GCS scores in these patients, as noted in previous studies [1, 4, 17] and also noted in our study. Also in our study, patients in bilateral group had significantly higher median number of associated intra-abdominal organ, head and chest injuries which denoted that the overall extent of injury was more in bilateral than unilateral injuries. So, the stormier course in hospital stay and worse final outcome in bilateral adrenal trauma population may also be secondary to the greater extent of injuries in these patients.

Castaldo et al. [17] specifically designed a study to explore whether adrenal injuries are predictive of adrenal insufficiency and concluded that while presence of adrenal injuries are a surrogate marker for more severe injury, both bilateral and unilateral adrenal injuries do not directly contribute to adrenal insufficiency. However in their study, only 1 out of 13 patients with bilateral adrenal hematomas had documented evidence of adrenal insufficiency while 10 out of 13 patients in our study had clinical episodes consistent with adrenal insufficiency as described. But we did not acquire the serum cortisol levels in our patients to look for adrenal insufficiency in our patients. Also while there were episodes of hyperkalemia in some of our patients with bilateral adrenal trauma, we did not note or follow-up the serial sodium and potassium levels in our patients. While lack of direct evidence of adrenal insufficiency in form of serial assessments of serum cortisol, sodium and potassium in our patients are potential limitations of our study, we believe that even if recorded, these levels would have been inconclusive of the true internal milieu as our patients were given exogenous steroids and electrolytes infusions. The more definitive proof of adrenocortical insufficiency as obtained by cosynotropin stimulation test is generally not feasible in trauma patients [21]. But there is scope for further large-scale evaluation of the differences between unilateral and bilateral adrenal trauma patients which unfortunately is also limited by the relative rarity of bilateral adrenal trauma.

To conclude, we had a substantial subset of patients detected to have bilateral adrenal injuries on CT in our study. Patients with bilateral adrenal hematomas fared worse than unilateral adrenal trauma due to a variety of other contributory factors and were clinically associated with more severe injuries, higher morbidity and mortality. Thus patients with adrenal injuries should be carefully monitored and pre-emptive supplemental or replacement steroid therapy with or without vasopressor support should be considered in patients with bilateral injuries.

## Consent

Written informed consent was obtained from the patient for the publication of this report and any accompanying images.

## References

[1] Stawicki SP, Hoey BA, Grossman MD, Anderson HL, Reed JF (2003). Adrenal gland trauma is associated with high injury severity and mortality. Curr Surg.

[2] Mehrazin R, Derweesh IH, Kincade MC, Thomas AC, Gold R, Wake RW (2007). Adrenal Trauma: Elvis Presley Memorial Trauma Center Experience. Urology.

[3] Burks DW, Mirvis SE, Shanmuganathan K (1992). Acute adrenal injury after blunt abdominal trauma: CT findings. AJR Am J Roentgenol.

[4] Rana AI, Kenney PJ, Lockhart ME, McGwin G, Morgan DE, Windham ST (2004). Adrenal Gland Hematomas in Trauma Patients. Radiology.

[5] Gabalshehab L, Alagiri M (2005). Traumatic adrenal injuries. J Urol.

[6] Iuchtman M, Breitgand A (2000). Traumatic adrenal hemorrhage in children: an indicator of visceral injury. Pediatr Surg Int.

[7] Schwarz M, Horev G, Freud E, Ziv N, Blumenfeld A, Steinberg R, Kornreich L (2000). Traumatic adrenal injury in children. Isr Med Assoc J.

[8] Roupakias S, Papoutsakis M, Mitsakou P (2011). Blunt adrenal gland trauma in the pediatric population. Asian J Surg.

[9] Udobi KF, Childs EW (2001). Adrenal Crisis after Traumatic Bilateral Adrenal Hemorrhage. J Trauma.

[10] Wolverson MK, Kannegiesser H (1984). CT of bilateral adrenal hemorrhage with acute adrenal insufficiency in the adult. AJR Am J Roentgenol.

[11] Ikekpeazzu N, Bonadies JA, Sreenivas VI (1996). Acute bilateral adrenal hemorrhage secondary to rough truck ride. J Emerg Med.

[12] Pinto A, Scaglione M, Guidi G, Farina R, Acampora C, Romano L (2006). Role of multidetector row computed tomography in the assessment of adrenal gland injuries. Eur J Radiol.

[13] Sinelnikov AO, Abujudeh HH, Chan D, Novelline RA (2007). CT manifestations of adrenal trauma: experience with 73 cases. Emerg Radiol.

[14] Sivit CJ, Ingram JD, Taylor GA, Bulas DI, Kushner DC, Eichelberger MR (1992). Posttraumatic adrenal hemorrhage in children: CT findings in 34 patients. AJR Am J Roentgenol.

[15] Ikeda O, Urata J, Araki Y, Yoshimatsu S, Kume S, Torigoe Y, Yamashita Y (2007). Acute adrenal hemorrhage after blunt trauma. Abdom Imaging.

[16] Pinto A, Scaglione M, Pinto F, Gagliardi N, Romano L (2003). Adrenal injuries: spectrum of CT findings. Emerg Radiol.

[17] Castaldo ET, Guillamondegui OD, Greco JA, Feurer ID, Miller RS, Morris JA (2008). Are adrenal injuries predictive of adrenal insufficiency in patients sustaining blunt trauma?. Am Surg.

[18] Francque SM, Schwagten VM, Ysebaert DK, Van Marck EA, Beaucourt LA (2004). Bilateral adrenal haemorrhage and acute adrenal insufficiency in a blunt abdominal trauma: a case-report and literature review. J Emerg Med.

[19] Baccot S, Tiffet O, Bonnot P, Perrot L, Cuilleret J (2000). Bilateral post-traumatic adrenal hemorrhage. Report of a case with acute adrenal insufficiency. Ann Chir.

[20] Sevitt S (1955). Post-traumatic adrenal apoplexy. J Clin Pathol.

[21] Gannon TA, Britt RC, Weireter LJ, Cole FJ, Collins JN, Britt LD (2006). Adrenal insufficiency in the critically ill trauma population. Am Surg.

